# Two-Dimensional Silicon Carbide: Emerging Direct Band Gap Semiconductor

**DOI:** 10.3390/nano10112226

**Published:** 2020-11-09

**Authors:** Sakineh Chabi, Kushal Kadel

**Affiliations:** Department of Mechanical Engineering, University of New Mexico, Albuquerque, NM 87131, USA; kushalkadel@unm.edu

**Keywords:** silicon carbide, two-dimensional materials, semiconductor, optoelectronics

## Abstract

As a direct wide bandgap semiconducting material, two-dimensional, 2D, silicon carbide has the potential to bring revolutionary advances into optoelectronic and electronic devices. It can overcome current limitations with silicon, bulk SiC, and gapless graphene. In addition to SiC, which is the most stable form of monolayer silicon carbide, other compositions, i.e., ${\mathrm{Si}}_{x}C_{y},$ are also predicted to be energetically favorable. Depending on the stoichiometry and bonding, monolayer ${\mathrm{Si}}_{x}C_{y}$ may behave as a semiconductor, semimetal or topological insulator. With different Si/C ratios, the emerging 2D silicon carbide materials could attain novel electronic, optical, magnetic, mechanical, and chemical properties that go beyond those of graphene, silicene, and already discovered 2D semiconducting materials. This paper summarizes key findings in 2D SiC and provides insight into how changing the arrangement of silicon and carbon atoms in SiC will unlock incredible electronic, magnetic, and optical properties. It also highlights the significance of these properties for electronics, optoelectronics, magnetic, and energy devices. Finally, it will discuss potential synthesis approaches that can be used to grow 2D silicon carbide.

## 1. Introduction

The discovery of monolayer silicon carbide will accelerate various technological innovations in the post-Moore era. As a wide bandgap semiconducting material with high thermal capability, SiC is a leading material for high-power electronics and high- temperature applications. However, due to quantum confinement and surface effects, 2D SiC offers tremendous unprecedented properties, which are absent in bulk SiC materials [1,2,3,4,5].

Unlike graphene, which is a pure one atom carbon material, 2D silicon carbide is a heteroatomic material that may exist in a variety of compositions and hence structures i.e., ${\mathrm{Si}}_{x}C_{y}$ e.g., SiC, ${\mathrm{SiC}}_{3}$, ${\mathrm{SiC}}_{7}$, among others. Further, unlike graphene which can be exfoliated from bulk graphite *via* mechanical exfoliation, the synthesis of single-layer SiC is one of the most challenging and tricky syntheses among 2D materials, demanding deep understanding of the atomic structure of the bulk SiC and its crystal structures. The main challenge is that bulk SiC is not a layered van der Waals material. It is a covalently bonded material with *sp*^3^ bonding between carbon and silicon along the c axis. As such, the formation of a monolayer silicon carbide requires phase transformation from $sp^{3}$ to $sp^{2}$. These structural challenges lead to the following fundamental questions: How will hexagonal 2D SiC be isolated from the tetrahedrally coordinated bulk SiC if the top-down approach would be adopted? Additionally, how easy would be the phase transformation from $sp^{3}$ to $sp^{2}$? Or at what thickness does the transformation take place? Furthermore, how stable is 2D SiC? Does it have ideal planar structure or slightly buckled form? And more importantly is 2D SiC stable in air? Or is it highly reactive?

Herein, we address these outstanding questions by reviewing the latest efforts and progress in the field of 2D silicon carbide, focusing on the structure, properties, and potential applications of these emerging 2D materials. This paper is organized as follows. The first section will provide a fundamental understating of the structure of 2D silicon carbide. Then, the key properties of 2D SiC will be discussed. Finally, we will outline future opportunities and challenges.

## 2. The Structure of 2D Silicon Carbide

**Figure 1:**
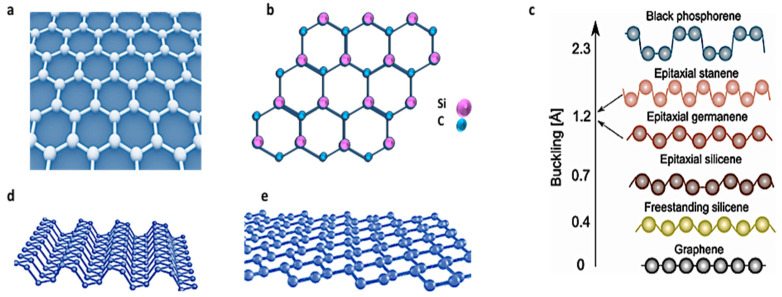
Schematics of crystal structure of graphene (**a**) and 2D SiC (**b**). Reported buckling for different 2D materials. (**c**) Unlike silicene and black phosphorous (BP), 2D SiC is 100% planar. (**c**) is reproduced from ref [15]. Atomic Structure of 2D black phosphorus (**d**). Credit: Institute for Basic Science. Atomic structure of silicene (**e**). Reprinted by permission from Nature [16], Copyright (2015).

Structurally, 2D SiC is predicted to have a graphene-like honeycomb structure consisting of alternating Si and C atoms. In the monolayer SiC, the carbon and silicon atoms will bond through $sp^{2}$ hybrid orbitals to form the SiC sheet, Figure 1. Various research groups have investigated the stability of planar 2D SiC, and all these studies have confirmed that 2D SiC is energetically stable and has a 100% planar structure with inherent dynamic stability [6,7,8]. They found that, while there may be a competition between $sp^{2}$ hybridization preferred by C in its planar form and $sp^{3}$ preferred by Si, the ground state of 2D SiC is completely flat, as planar 2D SiC has the lowest energy [9,10,11].

The predicted planarity feature is very important, and it contributes significantly to the development of several unprecedented properties. In fact, except graphene and hexagonal boron nitride, h-BN, most of the explored 2D materials, do not have stable planar structures. Instead, they stabilize their monolayer structures via i.e., a mix of $sp^{3}/sp^{2}$ bonding i.e., buckling. For instance, buckling values of 0.44 Å, 0.65 Å and 2.3 Å have been reported for silicene, germanene, and black phosphorous (BP), respectively [11,12,13,14,15]. In the case of silicene, it was even suggested that one approach to reducing buckling level is to use alternate atoms instead of pure silicon. Thus, given that 2D SiC could be considered as a heteroatomic form of silicene, it is reasonable that it has a stable planar structure.

As shown in Figure 1e, side view of silicene, as a result of mixed hybridization, *sp*^3^ and *s*p^2^, the bonds between adjacent atoms of the silicene lattice are buckled, resulting in a layer that is not completely flat. It is of note to mention that like graphite, black phosphorous is a van der Waals layered material. The buckling character affects the properties of 2D buckled materials significantly. For instance, because silicene does not have a perfect planar structure, it has a lower intrinsic electron/hole mobility than graphene.

Successful application of any 2D materials depends firstly on the material’s chemical and environmental stability. For instance, 2D BP-based devices have yet to be realized, because 2D BP suffers from a lack of environmental stability [17]. In the case of silicon carbide monolayer, there has been some skepticism about the stability of planar monolayer SiC due to the high reactivity of Si=C bonding. Although this reasoning is understandable, since $sp^{2}$ bonding is not the preferred configuration for bulk SiC, the case at ultralow thickness is different. The stability of the $sp^{2}$ hybrid in monolayer SiC can be attained in several potential ways.

In fact, 2D SiC is not the first structure that has Si=C double bonding, as such the idea of planar 2D SiC is not without foundation. A variety of Si=C containing compounds, known as “silenes’’ have been reported in the past [18,19,20]. The stability of Si=C bond in such materials is attributed, to a great extent, to the depolarization of the Si=C double bond as a result of the electronic effects of the substituents on the double bond. Such substitutes reduce the natural polarity of the Si=C bond through effects on both the positive charge density at silicon, $\delta^{+}$, and negative charge density, $\delta^{-}$, at carbon [21,22]. In addition to the reduced polarity, steric protection due to bulky substituents, and aromaticity conjugation may also contribute to the stabilization of $sp^{2}$ bonding between Si and C [20,23,24,25,26,27].

Similarly, the stability of monolayer 2D SiC can be discussed in terms of electronic effects and surface depolarization. Freeman et al. [28] used density functional theory (DFT) calculations to study phase transformation and stability in ultrathin wurtzite SiC, ZnO, GaN, BeO, and AIN films. They predicted that when wurtzite SiC or ZnO structures are thinned down to few atomic layers, they adopt graphitic like structure in which the atoms are threefold coordinated. This prediction was then confirmed experimentally for ultrathin films such as ZnO films [29], and AlN [30]. Uncompensated polarity and the transformation to graphitic-phase has also been reported for MgO ultrathin films [31,32]. Thus, although a significant gap of knowledge exists about the certain thickness at which phase transformation occurs, we do know that monolayer silicon carbide is stable and has a planar structure without any buckling. However, since bulk SiC has tetragonal $sp^{3}$ bonding, a phase transformation from $sp^{3}$ to $sp^{2}$ is required as single layer SiC is isolated from bulk SiC. As a result of phase transformation, the length of the Si-C bonds gets reduced, from 1.89 to 1.79 Å, and the bond angle increases from 109 to 120 degrees. Depending on the configuration and stacking sequence, different values might be achieved for interlayer distances [33]. Additionally, the phonon dispersion of 2D SiC has also been investigated by various research groups [2,9,34], and the calculated phonon spectra show no imaginary frequencies, indicating the stability of 2D SiC. Table 1 summarizes the structural characteristics of 2D SiC vs. other related materials.

**Figure 2:**
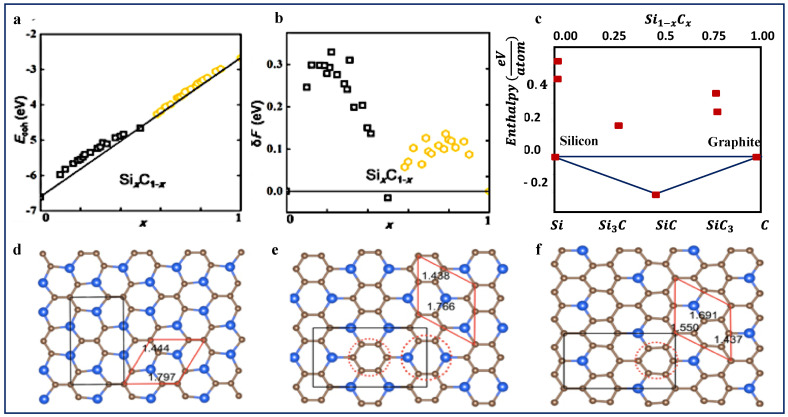
Cohesive energies, formation energies, and structures of SixCy materials. (**a**,**b**) Predicted cohesive energies and formation energies of 2D SixCy. Reprinted with permission from [9]. Copyright (2015) American Chemical Society. (**c**) predicated formation enthalpies of bulk $Si_{x}C_{1-x.}$ Reprinted with permission from [36]. Copyright (2013) American Chemical Society. As shown in (**a**), all investigated ${\mathrm{Si}}_{x}C_{y}$ structures have negative cohesive energy, meaning that they are energetically favorable. These values also suggest that if these structures can be made, they will resist and will not break into graphene, or any other materials. However, as shown in b and c, only Si_0.5_C_0.5_ has a negative formation energy, an indication of high stability. (**d**–**f**) predicted structures for SiC_2_,SiC_3_,and SiC_7_ respectively. Republished from [49], with permission from IOP. As shown, unlike 2D SiC which has average bond length of 1.79 Å, two bond lengths exist in the presented structure, one belongs to C-C, and the other to Si-C. It should also be noted that more than a hundred structures have been predicted for 2D ${\mathrm{Si}}_{x}C_{y}$, and the presented structures here only represent a few examples of the most studied structures.

As a binary compound, 2D silicon carbide may exists in a variety of compositions i.e., 2D SixCy. As such, thermodynamic and kinetic stability of 2D SixCy has been investigated by various theoretical studies [9,43,44,45,46,47]. Shi et al. used the cluster expansion method to investigate the stability of 2D honeycomb ${\mathrm{Si}}_{x}C_{1-x}$ structures, Figure 2 [9]. As shown, all compositions have positive formation energies except ${\mathrm{Si}}_{0.5}C_{0.5}$, i.e., SiC, which has negative formation energy and lies below the straight lines connecting the two phases boundaries, x = 0 and 1. Negative formation energy is an indication of high stability. These findings agree very well with all other theoretical studies [6,43,48], confirming that SiC is the most stable stoichiometry in the whole Si−C system in its ground state. However, as shown, all other compositions have relatively small formation energies, less than 100 eV for some compounds, and negative cohesive energy, suggesting that these structures are not completely unstable. Instead, they are metastable, and perhaps could be stabilized in some ways in the future.

These results are very similar to the findings from bulk silicon carbide. In a related study [36], Hoffmann group studied the stability of ${\mathrm{Si}}_{x}C_{y}$ phases at P = 1 atm in bulk materials. They found that although the simplest stoichiometric composition, i.e., SiC is the most stable phase, both Si3C and SiC3 are dynamically stable. Meaning although they are less stable than SiC, if they could be made, they would persist, as the activation energies for transforming them to SiC, C or Si is very large. More importantly, they found a relatively high stability of one type of graphene-like structure for SiC_3_ structures [36]. Interestingly, Zhao et al. [43] studied the stability of 2D SiC_3_, and 2D Si_3_C and similar results have been reported. Both 2D Si_3_C and 2D SiC_3_ are predicted to be topological insulators.

Additionally, it has been reported that carbon-rich SixCy compounds e.g., SiC_2_ or SiC_3_, are more stable than Si-rich compounds in their graphene-like structure. For example, using the global particle-swarm optimization algorithm and DFT calculations, Zhou et al. confirmed the thermodynamic stability of graphene-like SiC_2_ with an appropriate direct band gap [36]. In a similar study, Ref [50] studied and compared the stability of Si*_2_*C and SiC*_2_* compounds and found that graphene-like Si*_2_*C sheet has a higher formation energy, and thus unstable. Similar to bulk material, Si-rich 2D composites e.g., Si*_2_*C and Si*_3_*C are more stable in a diamond like 4 coordinated structures [36,50]. In addition to the structural differences, the key properties of 2D SixCy is determined by the Si/C stoichiometry as well. As a result of different composition, 2D silicon carbide could show a broad range of electronic and optical properties.

Regardless of the composition, alloying carbon and silicon atoms in such a planar two-dimensional binary system is very interesting, as this new 2D system offers a high level of capabilities and flexibilities, and functionalities, which are impossible in other closely related materials such as graphene or silicene. The next sections will provide a detailed discussion about the properties of 2D silicon carbide materials. First, electronic properties will be discussed.

## 3. Electronic Properties of 2D Silicon Carbide

Key properties of 2D SiC have been investigated using various density functional theory (DFT) approximations: Local-density approximations (LDA), generalized gradient approximations (GGA), GW corrections, Perdew, Burke, and Ernzerhof (PBE), and Heyd–Scuseria–Ernzerhof (HSE06) methods.

**Figure 3:**
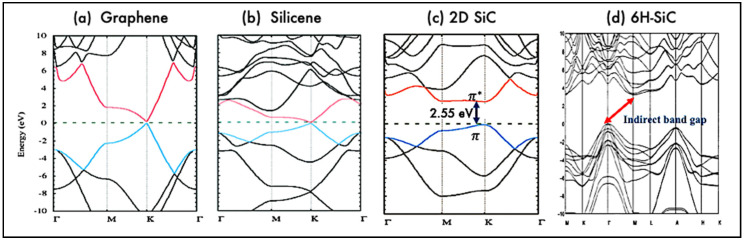
Electronic band structure of graphene, silicene, 2D SiC (**a**–**c**). Republished from [56] with permission from RSC. Electronic band structure of bulk 6H-SiC (**d**). Reproduced from [59] with permission from AIP publishing. Unlike graphene and silicene, 2D SiC have a band gap of about 2.55 eV (based on density functional theory (DFT)) due to its ionic nature. Further, 2D SiC has a direct band gap which is in contrast to indirect band gap in bulk SiC.

The electronic properties of 2D silicon carbide materials are determined basically through their electronic band structure. Figure 3 presents the band structures of hexagonal SiC monolayer, bulk 6H SiC, graphene, and silicene. As shown, both graphene and silicene are zero bandgap materials and in both materials, the valance and conduction bands, π and π*, meet at a single point at the Fermi level. However, hexagonal 2D SiC has a respectful band gap. The band gaps opening in 2D SiC is related to the electronegativity differences between Silicon and Carbon atoms, which would induce electron transfer from valance electrons of Si to the nearest C, so band gap emerges [34,41,51].

Theoretical calculations show that monolayer SiC is a direct bandgap semiconductor, that is in contrast with the indirect nature of the band gap in bulk SiC. Based on density functional theory, monolayer SiC has a direct band gap of 2.55 eV [37,52,53,54,55]. However, the calculated band gap increases to a higher value in the range of 3–4.8 eV when computed with GW quasiparticle corrections, GLLB-SC and other approximations [4,52,55,56,57]. The indirect-direct band gap transition characteristic in 2D SiC, is similar to the previously reported feature in other 2D materials such as 2D transition metal dichalcogenides (TMDs). This transition is attributed to the lack of any interlayer interactions in the TMDs monolayer [58]. It is of note to mention that TMDs are van der Waals layered materials like graphite. As such they can easily be fabricated via mechanical exfoliation.

Electronic properties of 2D silicon carbide depend strongly on the number of layers, as well as the atomic ratio between carbon and silicon in ${\mathrm{Si}}_{x}C_{y}.$ Although there is a significant gap of knowledge about the electronic properties of few layer and multilayer SiC, the band structure of few layer SiC is expected to experience significant deviation from that of bulk SiC. Depending on the stacking sequences, e.g., AB, ABC, different band structures and thus, properties might be attained.

It was also reported that unlike monolayer SiC which has a direct bandgap, multilayer SiC has been found to have an indirect bandgap [37,60,61]. However, indirect-direct band gap crossover, similar to the case of MoS_2_ and other TMDs, is possible for few layer SiC. This crossover, which reaches its limit in monolayer SiC, is attributed to the reduced dimensionality and electronic confinement in the direction perpendicular to the c axis. The bandgap of few layer silicon carbide is expected to decrease as the number of layers increases. The latter can be attributed to the reduced dielectric screening in monolayer silicon carbide [58,62].

The size of the bandgap and carrier mobility in 2D ${\mathrm{Si}}_{x}C_{y}$ showed a strong dependency on the atomic ratio between carbon and silicon [34,46,63,64]. Ref [9] used first-principles calculations combined with the cluster expansion method to study the structural and electronic properties of the monolayer ${\mathrm{Si}}_{x}C_{1-x}$. They found that most of the 2D ${\mathrm{Si}}_{x}C_{1-x}$ materials exhibit semiconducting properties, while only two structures, ${\mathrm{Si}}_{0.17}C_{0.83}$ and ${\mathrm{Si}}_{0.83}C_{0.17}$, are semimetallic, akin to graphene and silicene. Zhou et al. [50] reported that hexagonal SiC_2_ is a semiconductor with a direct band gap of 1.09 eV(HSE06) or 0.6 eV (PBE). Hexagonal SiC_2_ shows thermal stability up to 3000 K. Strained pentagonal 2D SiC_2_ is expected to have a very high hole mobility of $1.14\times 10^{6}\;{\mathrm{cm}}^{2}V^{-1}s^{-1}$, exceeding that of graphene [44]. Ref [43] used first-principles calculations combined with a tight-binding model to study the electronic properties of ${\mathrm{SiC}}_{3}$, and ${\mathrm{Si}}_{3}C$. They reported that both materials are topological insulators, and have Dirac cones centered at the K and K′ points.

The formation of Dirac cones is attributed to the preservation of the π-conjugate orbitals and hexagonal symmetry in both ${\mathrm{SiC}}_{3}$ and ${\mathrm{Si}}_{3}C$ structures as a result of their atomic arrangement. Similar to graphene, these materials might show the quantum-Hall effect. In a related study, Ref [63] used density functional calculations and particle-swarm optimization to investigate the electronic properties of ${\mathrm{SiC}}_{3}$. They reported that depending on the location of Si atoms, 2D ${\mathrm{SiC}}_{3}$ may behave as a semiconducting or semimetal material. If Si atoms are located in a meta position, then ${\mathrm{SiC}}_{3}$ sheet is a direct bandgap material. If silicon atoms are placed in a para position, then ${\mathrm{SiC}}_{3}$ is a zero-bandgap semimetal with distorted Dirac cones.

Electronic properties of 2D ${\mathrm{SiC}}_{6}$ and ${\mathrm{SiC}}_{7}$ have also been investigated [46,47]. A bandgap of 0.73 eV, and high electron mobility of $10^{4}\;{\mathrm{cm}}^{2}V^{-1}s^{-1}$, along the [$\bar{1}11]$ direction, have been reported for $SiC_{6}$ [47]. The carrier mobility of ${\mathrm{SiC}}_{6}$ can be further increased *via* mechanical strain. On the other hand, it has been reported that graphene like ${\mathrm{SiC}}_{7}$ is a semiconductor material with a direct band gap of 0.76 eV (PBE) or 1.13 eV (HSE06) [46]. Ref [65] studied electrical conductivity of SiC_3_ and SiC_7_. σ/τ values of $5\times 10^{18}\;\left(\Omega \mathrm{mS}\right)$^−1^ and $1\times 10^{16}\;\left(\Omega \mathrm{mS}\right)$^−1^ have been reported for SiC_3_ at 200 K, and SiC_7_ at 600 K respectively. Here, σ is the electrical conductivity and τ is the time relaxations. Additionally, it has been reported that as the temperature increases, σ/τ decreases for SiC_3_, and increases for SiC_7_.

The effects of the edge structure have also been investigated [54,66]. It has been reported that the armchair SiC nanoribbons are nonmagnetic semiconductors, while the zigzag nanoribbons are magnetic metals [56]. The band gap in armchair SiC nanoribbons can be tuned via hydrogen passivation. It was also reported that half-metallic zigzag edges can be turned metallic or semiconducting via functionalization with O or S atoms, respectively.

Additionally, the electronic properties of 2D SiC are highly affected by the defect level. For instance, local Dirac cones may form in 2D SiC, similar to graphene, as a results of C=C and Si=Si pairing [64]. C=C defects can be introduced into Si/C systems during the synthesis. In additional to defects, mechanical strain and chemical doping can also be used to tune the electronic properties of 2D SiC [60,67,68,69]. Not only the size of the bandgap, but even its nature can be changed from direct to indirect via mechanical strain [70]. Basically, since the charge distribution varies as the bond length changes, thus strain can play a critical role in tuning the electronic and optical properties of layered SiC [70].

As discussed, 2D silicon carbide materials, ${\mathrm{Si}}_{x}C_{y}$, benefit from highly tunable electronic properties. The band structure can be controlled via playing with Si/C composition, mechanical strain, and defects. This modifiability is of significant importance as it will enable the use of 2D silicon carbide for various applications.

## 4. Optical Properties of 2D Silicon Carbide

Unlike bulk silicon carbide which is an indirect semiconductor with weak absorption and light emitting characteristics, 2D silicon carbide has very rich optical properties such as strong photoluminescence, and excitonic effects, as a result of its direct bandgap and quantum confinement effects. The optical absorption spectra of 2D silicon carbide are shown to vary depending on light polarization, number of the layers, and Si/C ratio in ${\mathrm{Si}}_{x}C_{y}$ structures.

**Figure 4:**
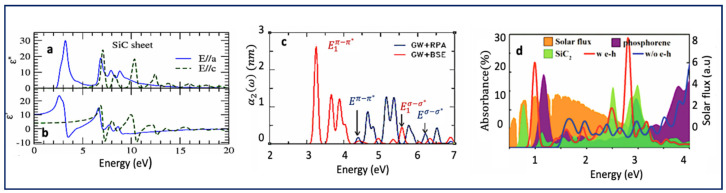
Optical properties of 2D silicon carbide materials. (**a**,**b**) Dielectric functions vs. photon energy in a single SiC sheet. The imaginary (**a**) and real (**b**)parts of the dielectric function for the electric field parallel (blue line) and perpendicular (green line) to the SiC sheet [53]. (**c**) Optical absorption spectra of monolayer SiC using GW+RPA (blue) and GW+BSE (red) calculations [4]. $\alpha_{2}$ is related to the imaginary part of the dielectric and it is imaginary part of the polarizability per unit area for a SiC sheet [4]. (**d**) Optical spectra of SiC_7_(orange, red and blue) vs. SiC_2_(green) and phosphorene(purple). Republished with permission of RSC, from [46] permission conveyed through Copyright Clearance Center, Inc.

In terms of light polarization, 2D SiC has highly anisotropic optical properties [4,53,71]. As shown in Figure 4a,b, dielectric function, which is a measure of light absorbance, show strong dependency on the light direction, and whether it is parallel or perpendicular to the SiC sheet base plane.

The complex dielectric is a function of frequency and it is: $\varepsilon \left(\omega \right)=\varepsilon^{\prime }\left(\omega \right)+i\varepsilon^{\prime \prime }\left(\omega \right)$, in which, $\varepsilon^{\prime }$ is the real and $\varepsilon^{\prime \prime }$ is the imaginary part [53,71]. The imaginary part of the dielectric function monolayer SiC is plotted in Figure 4a. As shown, under parallel direction, strong absorption peaks have been predicted in the low energy region 2–5.5 eV. On the other hand, under perpendicular electric field (parallel to c axis), absorption peaks are blue-shifted, and no absorption is expected below 5 eV [4,53,71]. Unlike parallel light, which results in strong/interesting optical properties, perpendicular light causes no significant optical absorption characteristics in the low photon energy region, i.e., below 6 eV. The latter is attributed to the weak dynamical screening under perpendicular light incident and the depolarization effects in that orientation [4,34,53]. Further, depending on the orientation of the polarized light, different transitions have been predicted. When the polarized electric field is parallel to the base plane, only π→π∗ and σ→ σ∗ transitions are allowed in low energy region. For perpendicular directions, only π→σ ∗ and σ → π∗ are expected [53].

Basically, as a result of quantum confinement, 2D SiC shows strong light-matter interactions, thus optical properties of 2D SiC are dominated by excitons which are composed of strongly correlated electron–hole pairs of the systems. However, different calculations have been used by scientists to predict the optical properties of 2D SiC. As such, depending on the approximation used, various results have been reported [4,34,46,50,53,72].

While some studies simply ignore electron–hole (e-h) interactions in their calculation, others do include it and as such different results were obtained. Hsueh et al. studied the effects of excitons on optical properties of monolayer SiC, and the results are presented in Figure 4c. They used GW+ RPA and GW+ BSE methods to investigate the effects of excitons [4]. Unlike the GW+ RPA method which does not include exciton effects in the calculation, the GW+ BSE method does include electron–hole interaction in the calculation of the optical properties of monolayer SiC. The first prominent peak located at 3.5 eV corresponds to the π and π* states transitions. As shown, electron–hole interaction dramatically changes the monolayer absorption.

The low-energy features in the energy range from 3.0 to 5.0 eV are dominated by π → π∗ transitions near the K point, whereas the pronounced absorption peak at 5.83 eV is mainly due to the σ → σ ∗ transition at the zone center. As shown in Figure 4c, the electron–hole interaction (red graph) not only triggers a red shift of the onset optical transition energies but also modifies their relative absorption intensities.

Figure 4d compares optical absorption properties of SiC_2_ with SiC_7_ and phosphorene. Compared to both SiC_2_, and phosphorene (single layer black phosphorus), SiC_7_ has stronger absorption in near infrared and visible photon ranges. Further, and as expected, absorption spectra obtained using approach includes electron–hole interaction, differ significantly from their independent particle counterparts. Introducing many-body effects, and specifically electron–hole interaction, gives rise to bound exciton states that strongly affect spectral properties [4,46,53,73]. These results strongly confirm the importance of including many-body effects and going beyond the independent particle model for those systems.

2D SiC has theoretical exciton binding energy of about 0.5–3 eV, which is more than one order of magnitude larger than the binding energy seen in bulk SiC [4]. This difference shows clearly that the reduced dimensionality of monolayer SiC, confines the quasiparticles, resulting in strong interactions between the electron and the hole wave functions and hence the electron–hole interaction. The weak dielectric screening in monolayer silicon carbide along with enhanced coulomb interaction, leads to the formation of tightly bound excitons that are stable at room temperature. On the other hand, both the surrounding layers, in multilayer SiC, and substrate [58] leads to an enhanced screening, which is expected to decrease both the exciton binding energy and the quasiparticle electronic band gap of monolayer silicon carbide. Such effects have been reported previously in other low dimensional materials such as 2D TDMs and carbone nanotube. Not only surrounding layers, but also substrate can increase the screening, resulting in reduced exciton binding energy and reduced band gap [58].

Optical properties of 2D silicon carbide, such as absorption flux, exciton binding energy, optical conductivity, are also strongly affected by the atomic ratio between carbon and silicon. Depending on the compositions, ${\mathrm{Si}}_{x}C_{y}$ materials have different band structures and thus band gap. As discussed earlier, among ${\mathrm{Si}}_{x}C_{y}$ materials, 1:1 stoichiometry, i.e., SiC is expected to have the largest band gap. It also has very large exciton binding energy and strongly coupled excitons. Other compositions are expected to have relatively smaller exciton binding energies [46,73]. For instance, 2D SiC_2_ has smaller exciton binding energy than monolayer SiC. This means that such an exciton is relatively readily can be dissociated into free electrons and holes [50]. Further, as shown in Figure 4d, depending on the assumption used in the calculation different absorption spectra can be obtained (red vs. blue). The light absorbance with electron–hole interaction (red line) shows absorption characteristics in the range of 0.7–3.0 eV. The first prominent peak corresponds to a bright exciton is located at 1.0 eV, showing great promises for solar devices.

Theoretical studies have also reported that 2D silicon carbide has strong nonlinear optical properties [72,74,75]. Ref [72] calculated the second harmony generation (SHG) spectrum of 2D SiC using a real-time first-principles approach based on Green’s function theory. They reported that SHG from a monolayer SiC is about one order of magnitude larger than that of standard nonlinear crystals. Such a strong SHG characteristics is attributed to the excitonic effects in 2D SiC. Nonlinear optical properties are of interest for nanoscale nonlinear frequency conversion devices. It is of note to add that SHG is a useful tool to study the number of layers or probe the structure of the edges in these materials [72].

The nonlinear optical properties in silicon carbide materials, are also affected by the atomic ratio between C and Si. For instance, it was reported that carbon-rich ${\mathrm{Si}}_{x}C_{y}$ materials, in bulk silicon carbide, have much enhanced nonlinear refractive index than Si-rich materials. This enhancement is related to the enhanced saturable absorbance in c-rich materials as a result of delocalized p-electrons [75]. Thus, depending on the composition and atomic configuration, 2D ${\mathrm{Si}}_{x}C_{y}$ materials might exhibit completely different nonlinear optical properties, and different mechanism are probably associated with these changes.

## 5. Magnetic Properties

There has been a great interest in understanding magnetism properties of 2D SiC and related material as well, as bulk SiC is considered one of the great candidates for magnetism and spintronic applications. Although perfect monolayer planar SiC was found to be a non-magnetic semiconductor [52,76,77], other forms of 2D SiC including defect-contained monolayer exhibits magnetism behavior [78,79]. Theoretical studies have found that the magnetic properties of 2D SiC can be tuned through: (i) doping, (ii) structural defects and (iii) mechanical strain, among others. 

One effective approach to tune the magnetic properties of 2D SiC is chemical doping, as this approach has been used successfully to tune the magnetic properties of several 2D materials. Magnetic moment in the doped materials arises from the asymmetric behavior of energy levels around fermi level in their resulting electronic structures. Variety of dopants including transition metals (TMs) [68,76,79,80,81] and non-magnetic metals (NMMs) [69] have been considered to tune magnetism behavior of 2D SiC. Among the two sites, C and Si, doping is found to be more favorable in Si-substituted sites, owing to the high structural stability of the resulting system [68,69,80]. For instance, 2D SiC exhibits magnetic behavior when Si atom is substituted by a Manganese (Mn) atom (total magnetic moment of 2.121 µB in 5 × 5 × 1 supercell) [68] and non-magnetic behavior when C atom is replaced by Magnesium (Mg) atom [69].

Among TM and NMMs dopants, TM dopants have been found to be more efficient in introducing magnetism into 2D SiC [52,68,69,80]. Thus, not only the position of the dopant, but also its nature, affects the magnetic properties of the doped 2D SiC. Ferromagnetic behavior has also been reported for electrically doped SiC monolayer [76]. Further, magnetic coupling between the dopant and SiC is also very important. Magnetic coupling is related to the hybridization of dopant and its neighboring atoms. For instance, depending on the dopant-dopant distance, 2D SiC may switch from antiferromagnetic to ferromagnetic [68,69,81].

Another efficient strategy to engineer magnetic properties of 2D materials is vacancy defects. This approach has been used successfully in manipulating magnetism and spin fluctuations in graphene. In 2D SiC, three types of vacancy defects have been studied; single C or Si vacancy, Si + C divacancy, and Si-C antisite defects in the monolayer [52,76,77,78].

It has been found that only silicon vacancy induces local magnetism into the system as a result of spin polarization around the defect sites. As a results of silicon vacancy, an unpaired C=C formed that leads to spin polarization and magnetic moment in monolayer SiC. However, the magnetic coupling between Si vacancy-induced local moments is very weak owing to the small energy difference between different spin configurations [77]. In both C and C+ Si vacancies, the materials remain non-magnetic. In this case, Si atoms surrounding the C vacancy form a weak bond, which finally leads to the formation of electron pairs. As such, no-spin polarization occurs in the system with C-vacancy due to structural readjustment [52,77]. Similarly, Si + C divacancy and Si-C antisite defects do not induce magnetic moment in the system [52].

Defects grown during the synthesis or surface defects may introduce some magnetism behavior into 2D SiC [76,78]. For instance, unpaired C vacancies could result in ferromagnetism behavior in monolayer SiC. In fact, room temperature ferromagnetic has been reported in 2D SiC as a result of vacancy defects [67]. These vacancies, i.e., unpaired carbon vacancies can be identified using Raman spectroscopy, as they show a peak around 2700 cm^−1^.

It has been also reported [78] that as the thickness of SiC nanosheets decreases, e.g., from 9–3 nm, the saturation magnetization increases. The observed magnetism could be related to defects with carbon dangling bond on the surface of nanosheets. Although grown-in defects may lead to ferromagnetism at room temperature in 2D SiC, there are many challenges involved in this scenario, including the lack of control over the density, type, or location of defects. Mechanical strain, can also be used to tune magnetic properties of these materials [60,82]. For instance, it has been found that the use of compressive strain, transfers 2D SiC from a semi-conductor to metal [82]. Similar switchable magnetism has been observed in Mn-doped 2D SiC as well [76].

Various research groups have also investigated the magnetic properties of SiC nanoribbons [54,83]. It has been found that unlike armchair edges that are non-magnetic semiconductor, zigzag edges are magnetic metals. Magnetism properties of zigzag nanoribbons are highly affected by the diameter of the nanoribbons, and it has been reported that zigzag SiC nanoribbons narrower than 4 nm possess half metallicity without applied external field or chemical doping [54]. Further, C-edged 2D SiC triangular nanoflakes based kagome lattice has been found to exhibit ferromagnetism at room temperature [84]. Another notable attempts to introduce magnetism in the monolayer SiC includes (i) O adatom adsorption [82] and (ii) direct electron doping controlled by electrolyte gating [76].

Indeed, as discussed earlier, 2D SiC possesses great potentials as a versatile magnetic material. Such a flexibility and modifiability e.g., acting as a ferromagnetic material at RT, is very useful for magnetic memories, magnetic storage and communications technology devices. However, these theoretical studies need to be followed by experimental research.

## 6. Mechanical Properties

Mechanical properties of any material are determined by its in-plane and out of plane atomic bonding. Silicon carbide is one of the strongest known materials because it bonds silicon and carbon through strong covalent bonds. Similar to bulk SiC, 2D SiC is a brittle material and a sudden drop in the stress at high strain has been predicted. However, unlike bulk SiC which is a covalently bonded material along both c-axis and a-axis, monolayer silicon carbide is an atom thick material, no c-axis. As such, 2D SiC is expected to have different mechanical properties than bulk SiC.

Theoretical studies indicated that 2D SiC has anisotropic mechanical properties [70,85,86]. Mechanical properties of 2D SiC, such as Young’s modulus, in-plane stiffness, and toughness, are strongly affected by the structure of the edges (armchair or zigzag) and their orientations, as well as the atomic ratio between Si and C in ${\mathrm{Si}}_{x}C_{y}.$ In terms of tensile properties, Young’s modulus of 2D SiC was found to be about 180 N/m in zigzag direction, and 175 N/m in armchair direction. This value is almost 53% that of graphene, which is 358 N/m. Fracture tensile stress of 2D SiC was also found to be about 21.0 N/m along the zigzag direction, which is almost half that of graphene. Reported strain at maximum stress is 23.2%, which is larger than that of graphene (20.3%) [70,85,86,87].

In the case of ${\mathrm{Si}}_{x}C_{y}$ monolayer, Young’s modulus is generally predicted to increases for carbon-rich compositions, providing that they are well dispersed. For instance, tensile Young’s modulus of 230 and 235 N/m have been calculated for SiC_3_ and SiC_7_ respectively, which are larger than that of 2D SiC [37,70,87]. Similar to Young’s modulus, larger fracture stress is predicted for majority of carbon-rich ${\mathrm{Si}}_{x}C_{y}$ structures [85,86,87].

The dependency of mechanical properties of ${\mathrm{Si}}_{x}C_{y}$ on x,y, is related to the arrangement of Si and C atoms in the lattice. As depending on the composition, different atomic bondings and configurations exist, resulting in different responses to the applied load.

2D SiC can withstand maximum compression stress of 85 N/m, which is much larger than its tensile fracture. This difference is due to anisotropy nature of its mechanical properties. 2D SiC is expected to have poison’s ratio of 0.29–0.32. [37,49,70] 2D SiC can be considered as a tough and stiff material. It has toughness and stiffness values of about 3.8 GPa and 166 J/m^2^ respectively. Graphene is only about 13.63% tougher than 2D SiC. Further, compared to many other 2D semiconducting materials such as silicene, AIN, and GaN, 2D SiC is much a stiffer material [37,70].

The effects of both uniaxial and biaxial stress, 20–140 N/m, on the mechanical behavior of 2D SiC has also been investigated, and 2D SiC demonstrated good structural integrity and mechanical stability under both uniaxial and biaxial stress [70].

The mechanical properties of 2D SiC might vary significantly depending on uniaxial stress, temperature, vacancy defects, vacancy concentration, strain rate, etc. [86,88,89]. For instance, a considerable mechanical degradation is expected as a results of defects. Not only the concentration of the defect but also its type has different impacts on the mechanical properties of these materials. For instance, some studies reported that single atom vacancy can degrade the mechanical properties of 2D SiC even more than bi-vacancy defects [86,88,89].

## 7. Device Applications

Having discussed fundamental properties of 2D silicon carbides, the next sections will discuss potential applications of this rapidly emerging material.

### 7.1. Optoelectronics

As a direct band gap material, 2D SiC has great potential for optoelectronic applications, such as light emitting diodes (LEDs), lasers, optical switches and solar cells. Monolayer silicon carbide also exhibits a tunable bandgap and a very bright emission which is a useful property for engineering the optoelectronic response for specific applications. This flexibility in band gap alteration enables the fabrication of light emitting devices such as LEDs covering the entire visible spectrum. In addition to its tunable band gap, monolayer silicon carbide has a large exciton energy as a result of enhanced electron–hole interaction and reduced dielectric screening, which is very useful for some optoelectronic applications. Large exciton binding energy leads to strong and long-lived excitons, thus making such materials indispensable for applications such as UV excitonic lasers [4]. This property is desired for LEDs, photo markers and excitonic solar cells.

Graphene can only absorb 2.3% of the normal incident light per monolayer, and as such its application as a photodetector material is limited. On contrary, 2D SiC exhibits 4.6% absorption of incident UV-light that can be boosted to 99.6% with gold plasmonic gratings [39,90,91]. Thus, it can be used as high-performance UV photodetector in high temperature, high power, and radiation-resistant applications.

2D SiC related materials such as ${\mathrm{Si}}_{x}C_{y}$ and quantum dots can enable additional technological applications. For instance, graphene-like SiC_2_ with a direct band gap of 1.09 eV can be used as a donor materials for excitonic solar cells [50]. Or it can be used in combination with other materials to enable a variety of highly efficient heterostructures. For instance a device based on a bilayer of g-SiC_2_/GaN or g-SiC_2_/ZnO may lead to achieving a tunable power conversion efficiency of about 12–20% [50]. Additionally, g-SiC_7_, has been reported to have superior sunlight optical absorbance over g-SiC_2_ in near infrared and visible photon ranges [46]. Further, as a biocompatible material, 2D SiC holds great promises for bioimaging and biosensor applications. Recently, 2D SiC quantum dots have also been used for cellular imaging and transport [92].

### 7.2. Electronics and Spintronics

As a one atom thick wide bandgap material, 2D SiC has huge potentials for electronic devices, especially high temperature, high-power, and high-frequency devices. The fact that monolayer silicon carbide is only one atom thick, give rise to two potential intriguing characteristics in SiC electronics, (i) reduced ohmic resistance as a result of reduced thickness and (ii) smaller, lighter nanoelectronics devices. Another advantage is that unlike bulk SiC, which has more than 250 polytypes, monolayer SiC does not have any polytype. The elimination of stacking sequences makes the device fabrication process less complicated. As discussed earlier, 2D silicon carbide is not limited to 2D SiC. It is on its own a big family of ${\mathrm{Si}}_{x}C_{y}$ structures. Depending on the composition, $2D\;{\mathrm{Si}}_{x}C_{y}$ may behave as semiconductor, with approximate bandgap ranging from 0.0 to 4.0 eV, topological insulator or semimetal. This flexibility further expands the realm of 2D SiC, allowing it to be used for both high and low frequencies devices.

2D SiC can be used to achieve a p-n junction. In fact, designing p-n junctions based on 2D materials is a significant advancement in modern technologies, because p-n junction is a fundamental building block of almost every electronic device. Due to the absence of an energy band gap and massless Dirac-like behavior of charge carriers, achieving a p-n junction with graphene and silicene is highly challenging. In contrast, 2D SiC has a respectful band gap and can be used for this purpose. Interestingly, p-n junction based on hexagonal 2D SiC is found to exhibit high rectification performance [93]. Its rectification ratio has been calculated to be ~10^5^ cal., relatively large compared to that of semiconductors such as Gallium arsenide (GaAs) and Silicon germanium (SiGe). Further, the dimensional reduction from bulk to 2D monolayer also allows a large reduction in resistance value, which can lead to high-speed switching operations in optoelectronic devices. The performance of these devices can be further controlled by designing them along zigzag or armchair direction.

2D SiC materials can also be used along with other 2D materials to make a variety of 2D materials-based heterostructure devices. For instance, graphene or h-BN materials can be used with 2D SiC when conductor or insulator (gate) are needed, respectively. Further still, compared to many 2D materials, except graphene and h-BN, monolayer 2D SiC has higher in-plane stiffness, Young’s modulus. Thus, it can be very beneficial for electromechanical devices.

2D SiC can be used for quantum spintronics as well. Spintronic refers to spin-based electronics. This rapidly evolving research field relies on spin-controlled electronic properties. Silicon carbide materials offer great promises to spintronic devices. For instance, spins associated with color centers in SiC can have long coherence times, compared to diamond [94,95]. However, the use of 2D SiC, instead of bulk SiC, offers an additional degree of freedom, and it may allow some control over the magnetic properties. As discussed earlier, 2D SiC has highly tunable magnetic properties. 2D SiC is a diamagnetic material in its perfect crystal structure but becomes a ferromagnetic material as a result of some defects such as silicon vacancy or chemical doping.

Some types of 2D silicon carbide materials such as Mn-implanted SiC show curie temperature (T_C_) greater than room temperature (RT) [76,96]. Such characteristics are very beneficial for spintronics. Moreover, analogous to graphene, C-edged 2D SiC triangular nanoflakes based kagome lattice has been found to exhibit ferromagnetism at RT, [84] which further confirms the potential of 2D SiC to act as a building block for novel magnetic structure and devices. Bipolar magnetism has also been reported for 2D SiC, which is beneficial for magnetic bipolar transistors. Magnetic bipolar transistors allow for magnetic and spin control of current amplification.

As a wide bandgap 2D material with high spin polarization and ferromagnetic properties at RT, 2D SiC and related materials e.g., quantum dot forms of 2D SiC can be very useful for spintronics. By exploiting the spin properties of 2D SiC, new functionalities and phenomena may be realized. Future research in 2D silicon carbide spintronics should also include understanding the spintronic behavior in 2D ${\mathrm{Si}}_{x}C_{y}$ materials and few-layer SiC such as 4H- 2D SiC. It is expected that carbon rich monolayer ${\mathrm{Si}}_{x}C_{y}$ shows even more promises than 2D SiC, for quantum technologies.

### 7.3. Chemical Sensing and Energy Applications

The potentials of 2D SiC for chemical sensing have been investigated by various research groups [9,97,98,99]. Compared to inert graphene, monolayer SiC is chemically more active, and it has abundant active sites for adsorption. 2D SiC can be utilized for chemical sensing such as humidity sensors [98], gas sensors [100,101] and adsorbents [102] for detecting and absorbing hazardous gases. For instance, Sun et al. demonstrated the ability of SiC nanosheet to quickly detect acetone, ethanol, methanol, and ammonia at a temperature of 500 °C [100]. Fermanzadeh et al. used SiC nanomaterials as an absorbent to capture harmful gases such as ozone, nitrogen dioxide, and sulfur dioxide [102]. SiC nanosheets can also be used to fabricate high temperature hydrogen sensors [101]. Moreover, SiC nanosheets-based humidity sensors are found to have high sensitivity to water in ambient temperature (16991.1 at 95% relative humidity) and a quick response/recovery time of 3 s [98]. The fast sensing property may be related to the reduced thickness which results in fast electron transfer. The sensing and absorbing properties of 2D silicon carbide material and related devices can further be improved via doping and appropriate substitutional impurities in the nanosheet [98,100,102]. For instance, SiC nanosheets demonstrated much better performance as a gas absorbent after doping them with Fe [102]. Silicon carbide nanosheets are also useful for several important catalysis reactions including oxygen reduction reaction (ORR) in fuel cells, and NO reduction [103,104,105,106].

Compared to Pt, SiC nanosheets exhibit superior ORR catalytic activity in alkaline media. Interestingly, they trigger the catalytic activity without CO poisoning effect, which is a major obstacle in Pt-based catalysts [105]. Additionally, 2D silicon carbide can be used as catalyst to facilitate NO reduction [104] and CO oxidation [103] reactions. Both reactions are critical to remove highly toxic gases from the environment. SiC monolayer can catalyst CO oxidation reaction with a small energy barrier of 0.65 eV [103].

Owing to their atomic thickness, 2D SiC is expected to have large surface area. This property is beneficial for capacitors, as they store charges physically on the surface without any electrochemical reactions. SiC nano sheets demonstrated high specific energy as well as high cycle life when used as electrode materials for GO-SiC supercapacitor. [99] Mesoporous SiC flakes have also been used for electrochemical capacitive energy storage in the past [107,108]. 2D silicon carbide materials, especially carbon-rich ${\mathrm{Si}}_{x}C_{y}$ materials showed great promises for lithium ion batteries. For instance, specific capacity values of 1520 mA h/g and 1286 mA h/g have been predicted for SiC_5_, and SiC_2_ respectively. Such a high specific capacity, along with the reduced dimensionality will result in both high energy density as well as high power density. It is of note to mention that the use of Si/C composites as anode materials for lithium-ion batteries has become prevalent in the last decade. However, one potential advantage of using 2D materials over bulk Si/C composites is the mechanical integrity of 2D materials.

## 8. Growth Approaches

Basically, two general approaches have been used to growth 2D materials: (i) top-down, and (ii) bottom up methods. Most of the discovered 2D materials have been synthesized via top-down approach. Top down approach refers to isolating 2D materials, single layer or few layers, by etching out crystal planes from bulk material. Some examples of top-down synthesis methods are mechanical exfoliation, liquid exfoliation, chemical etchings or a combination of both e.g., thermal oxidation etching and liquid exfoliation. This method has been used widely for the synthesis of graphene, graphene oxide, born nitride, MXenes, TDMs, and other layered 2D materials [109,110,111,112]. Usually, these exfoliation methods require the presence of layered structure in the bulk precursors, by which the weak van der Waals force among layers can be broken to create nanosheets or ideally monolayer structure. Unlike, graphite, boron nitride, and TMDs which are van der Waals layered materials, silicon carbide is not a layered material. Bulk SiC has strong *sp*^3^ covalent bonding along the c-axis, making it challenging to create a single layer SiC by relying on a single exfoliation route. Given that monolayer SiC has a planar $sp^{2}$ structure, phase transformation from $sp^{3}$ to $sp^{2}$ must take place as number of layer decreases.

On top of all above-mentioned challenges, SiC exists in more than 250 polytypes, making the growth process further complicated and the selection of the precursor extremely important. Given all these challenges, both top-down and bottom-up approaches have been used to grow SiC nanosheets. Liquid exfoliation of SiC powder has been used successfully to make SiC nano sheets with average size of 20–300 nm. Chemical preparation of multilayer SiC nanosheets have also been demonstrated using hydrothermal synthesis [7,78,92]. In the liquid exfoliation approach, it was found that both the size and thickness of SiC are profoundly affected by the centrifuge rate and exfoliation time. Although fabricating thinner nanosheets requires a longer exfoliation time and centrifuge, the latter compromises the nanosheets’ size. Thus, for a top-down approach to be successful here, it needs to be designed intelligently. The potential of the bottom-up approach and specifically the CVD method has also been explored [8,113,114,115]. Chabi et al. [8] were able to make SiC nanosheets, by adopting a two-steps synthesis procedure composed of a carbothermal synthesis of graphene and silicon followed by a liquid exfoliation step. The average size and thickness of the grown nanosheets was 2 μm, and 2 nm, respectively. Other potential synthesis methods include encapsulation, extreme hole injection on SiC surface and wet etching [2,35,116].

These promising earlier experimental studies reveal that although monolayer silicon carbide is yet to be synthesized, it is emerging in the near future. As indeed, there are many synthesis options in front of experimentalists. Successful growth/isolation of monolayer silicon carbide requires a smart selection of the following parameters: precursors, temperature, carrier gases, synthesis time, and the substrate. The substrate is very important, as it determines the symmetry, quality, crystal structure, and thickness of the deposited SiC film.

## 9. Concluding Remarks

2D silicon carbide shares many similarities with previously discovered 2D materials, and at the same time possesses abundant unique properties that cannot be found in any other 2D materials. Like graphene and h-BN it has stable planar structure, and unlike both, it is a direct bandgap material. Unlike black phosphorus and silicene, which form buckled structure, monolayer SiC is 100 % flat. Unlike TDMCs which have low carrier mobility, some forms of 2D silicon carbide can reach carrier mobility exceeding that of graphene. 2D silicon carbide is not a single new member in 2D family, it is on its own a very big family of ${\mathrm{Si}}_{x}C_{y}$ structures such as SiC_3_, Si_3_C, SiC_2_ and SiC_7_. Each member of this emerging big family challenges us with its previously unknown physics. The formation energy, configuration, structure, stability, and thus properties of these structures is highly affected by the atomic ratio of silicon and carbon and whether the structure is carbon- or silicon- rich. Given their tunable direct band gap in the rage of about 0.5–3 eV, the next generation of electronic and optoelectronic devices, can benefit largely from the discovery of 2D silicon carbide. Importantly, the properties of 2D ${\mathrm{Si}}_{x}C_{y}$ can be tuned by external stimuli such as electric field, strain, defects, and chemical doping. Moreover, 2D SiC offers tremendous potential for chemical sensing, quantum technologies and energy storage and conversion devices. These potentials and properties reveal how huge the emergence of 2D SiC will be.

Moving forward, we believe that there are four main directions to be pursued in the study of 2D silicon carbide: (i) The discovery of monolayer 2D SiC. Given that the stability of planar monolayer SiC has already been proved by the theory, we believe that monolayer silicon carbide can be synthesized by both top-down and bottom-up approaches and it will emerge in near future. (ii) Investigation of the phase transformation in few layer and multi-layer SiC and understanding the main mechanism behind this transition (iii) Utilization and exploitation of exotic optical, electronic and magnetic properties of SiC nanosheets and related structures. 2D silicon carbide possess a variety of exotic properties. It has a very large exciton binding energy as a result of very strong electron–hole interaction. This property, and other properties, need to be utilized and exploited in real devices. We already know how to make few layers SiC, SiC quantum dots, and other related structures. Like monolayer silicon carbide, few layers silicon carbide possess a range of impactful properties that deserves further attention and exploitation. For instance, SiC nanosheets can be used to develop a new generation of MOSFET, providing that their electronic properties such as electrical resistance and carrier mobility will be investigated experimentally first. Additionally, SiC nanosheets can be combined with other 2D materials to make novel heterostructure devices. (iv) Growth and application of 2D ${\mathrm{Si}}_{x}C_{y}$. 2D silicon carbide can be considered as a universal material. Because depending on the composition, ${\mathrm{Si}}_{x}C_{y}$ can exhibit the same properties as graphene, silicon or silicon carbide. As such, all these structures deserve further investigation and attention.

## Figures and Tables

**Table 1:** Structural characteristics of 2D SiC and other related materials.

Material	Bond Length (Å)	Lattice Constant (Å)	Configuration	Interlayer Distance (Å)	Refs
2D SiC	1.77–1.79	3.1	planar	varies	[2,10,35,36,37,38,39,40,41]
6H-SiC	1.89	3.08	*sp* ^3^	2.52	[2,36]
Graphene	1.42	2.46	planar	1.42	[2,41]
Silicene	2.27	3.8	buckled	varies	[9,37,42]
